# Progress in mitochondrial and omics studies in Alzheimer’s disease research: from molecular mechanisms to therapeutic interventions

**DOI:** 10.3389/fimmu.2024.1418939

**Published:** 2024-07-08

**Authors:** Zuning Liao, Qiying Zhang, Na Ren, Haiyan Zhao, Xueyan Zheng

**Affiliations:** ^1^ Department of Neurology, Fourth People’s Hospital of Jinan, Jinan, China; ^2^ Department of Internal Medicine, Jinan Municipal Government Hospital, Jinan, China; ^3^ Pharmacy Department, Jinan Municipal People’s Government Organs Outpatient Department, Jinan, China; ^4^ Department of Pharmacy, Qihe County People’s Hospital, Dezhou, China; ^5^ Department of Pharmacy, Jinan Second People’s Hospital, Jinan, China

**Keywords:** Alzheimer’s disease, mitochondrial dysfunction, multi-omics, molecular mechanisms, therapeutic strategies

## Abstract

Alzheimer’s disease (Alzheimer’s disease, AD) is a progressive neurological disorder characterized by memory loss and cognitive impairment. It is characterized by the formation of tau protein neurofibrillary tangles and β-amyloid plaques. Recent studies have found that mitochondria in neuronal cells of AD patients exhibit various dysfunctions, including reduced numbers, ultrastructural changes, reduced enzyme activity, and abnormal kinetics. These abnormal mitochondria not only lead to the loss of normal neuronal cell function, but are also a major driver of AD progression. In this review, we will focus on the advances of mitochondria and their multi-omics in AD research, with particular emphasis on how mitochondrial dysfunction in AD drives disease progression. At the same time, we will focus on summarizing how mitochondrial genomics technologies have revealed specific details of these dysfunctions and how therapeutic strategies targeting mitochondria may provide new directions for future AD treatments. By delving into the key mechanisms of mitochondria in AD related to energy metabolism, altered kinetics, regulation of cell death, and dysregulation of calcium-ion homeostasis, and how mitochondrial multi-omics technologies can be utilized to provide us with a better understanding of these processes. In the future, mitochondria-centered therapeutic strategies will be a key idea in the treatment of AD.

## 1 Introduction

AD is currently the most common form of dementia, and according to incomplete statistics, AD accounts for approximately 80% of dementia cases worldwide. As one of the most common neurodegenerative disorders in the world, AD is characterized by amyloid β (amyloid β, Aβ) plaques and neurofibrillary tangles of hyperphosphorylated tau protein, which are often accompanied by region-specific cerebral atrophy and a significant decrease in glucose metabolic utilization (1). Patients with AD exhibit typical clinical attention deficit disorder with extensive cognitive deficits, which severely affects their normal life (2). In recent years, an increasing number of elderly patients with AD have imposed a significant health and economic burden on people worldwide. Although many effective treatments exist for AD, they are limited to symptomatic relief and do not stop the progression of the disease or cure it (3). The etiology of AD is complex and involves the interplay of genetics, molecular biology, the environment, and other factors. The global prevalence of AD is rising, and the number of patients is expected to increase dramatically by 2050, with the number of people needing to care for them increasing accordingly (4). The incidence of AD is increasing globally. Therefore, accelerating the investigation of the pathogenesis of AD and searching for effective therapeutic targets to improve the clinical symptoms of patients are the key concerns of all future scientists (5).

Mitochondria, as the central part of energy metabolism in cells, play an important role in the normal physiological processes of all types of cells. In neuronal cells, mitochondria provide energy to cells through oxidative phosphorylation to maintain normal neuronal function. Once mitochondrial dysfunction occurs, the normal development and function of neurons will be abnormal, and various degenerative diseases will occur (6). The link between mitochondrial dysfunction and the development of AD, which is characterized by memory loss and cognitive dysfunction, amyloid-β plaque formation and abnormal phosphorylation of tau proteins, has attracted the attention of more and more researchers. Recent research evidence suggests that mitochondrial dysfunction not only drives the onset of AD, but also promotes its further progression (7). With the in-depth study of mitochondria and its multi-omics, including mitochondrial genomics, proteomics, metabolomics, epigenomics, etc., new ideas and perspectives have been provided to study the specific mechanism of mitochondrial role in AD (8). These high-throughput sequencing technologies enable researchers to fully understand the internal structure and heterogeneity of the mitochondria, while in-depth studies of mitochondrial DNA mutation mechanisms, mitochondrial gene and protein expression changes, and energy metabolic pathways can help us better elucidate the specific mechanisms of mitochondrial role in AD (9). In addition, the progress of mitochondrial multi-omics research will also improve the key targets for the treatment of AD in the near future, and promote the discovery and development of mitochondria-targeted drugs.

In this review, we focus on summarizing the specific roles of mitochondrial dysfunction in AD and how the use of mitochondrial multi-omics technology can reveal the mechanisms of these mitochondrial dysfunctions, and finally provide key targets and ideas for the treatment of AD. We aim to summarize a comprehensive perspective from molecular mechanisms to drug discovery centered on mitochondria in AD. In the future, mitochondrial multi-omics technology will reveal to a great extent how mitochondrial dysfunction drives the onset and progression of AD, and how mitochondria as a target can be targeted to treat AD and improve the quality of patients’ survival.

## 2 Mitochondrial dysfunction drives the onset of AD

### 2.1 Imbalance of mitochondrial dynamics in Alzheimer’s disease

Mitochondrial dynamics imbalance mainly refers to the dynamic imbalance between mitochondrial fission and fusion, which can directly lead to severe abnormalities in mitochondrial number and function, and directly induce the onset and progression of AD (10, 11). This dynamic imbalance is characterized by an increase in mitochondrial fission and a decrease in fusion, leading to mitochondrial fragmentation, which in turn causes an imbalance in mitochondrial energy production and oxidative phosphorylation. The Mitochondrial dynamics imbalance ultimately leads to severe neuronal damage, which in turn triggers attention deficit disorder (12). Mitochondrial fission is mainly regulated by dynamic-related protein 1 (dynamic-related protein 1, Drp1), which is recruited to the outer mitochondrial membrane by resident proteins such as mitochondrial fission 1 protein (fission 1 protein, Fis1) and mitochondrial fission factor (mitochondrial fission factor, Mff) to play a normal role in regulating mitochondrial fission. In AD-related studies, abnormal mitochondrial fission is usually positively correlated with elevated levels of Aβ and phospho-tau (phospho-tau, pTau) (13, 14). In AD, proteins such as Aβ and Tau further exacerbate the mitochondrial fission process by interacting with mitochondrial fission regulators, especially DRP1 and mitochondrial FIS1 (15, 16). Further studies revealed that Aβ induces increased S-nitrosylation of DRP1 and promotes the activity of mitochondrial fission-related enzymes, leading to increased fragmentation of the mitochondrial inner membrane and severe abnormal mitochondrial function (17, 18). Similarly, a study found that phosphorylation of the Ser616 site on Drp1 promotes the transport of Drp1 to the mitochondrial membrane and increases its aggregation at the membrane, thereby promoting mitochondrial fission. In addition, in AD, Aβ aggregation can lead to elevated levels of intracellular calcium ions and reactive oxygen species (reactive oxygen species, ROS), etc. Rising calcium ions and ROS induce the expression of kinases such as GSK-3 and ERK, which in turn promotes the process of phosphorylation of Drp1, and ultimately promotes mitochondrial fission. Mitochondrial fusion is mainly regulated by the fusion proteins Mfn1 and Mfn2 located in the outer mitochondrial membrane and by the fusion protein Opa1 located in the inner membrane (19, 20). In mice with Tau-ablated AD, a decrease in ROS, a decrease in fission, an increase in fusion, an inhibition of mitochondrial permeability transition pore (mitochondrial permeability transition pore, mPTP) and cyploheximide D, which promotes the normal functioning of mitochondria, and an enhancement of ATP production are observed. In AD, these fusions are regulated by the fusion of ROS, fission, and fusion. In AD, the expression and function of these fusion-regulated proteins are severely impaired, resulting in reduced mitochondrial fusion (21). The imbalance between mitochondrial fission and fusion affects not only the number and morphology of mitochondria, but also the integrity of mitochondrial DNA and mitochondrial function. One of the biggest effects is mainly the disruption of ATP production and regulation of intracellular calcium ions (22). In AD, another consequence of imbalanced mitochondrial dynamics is abnormal cellular mitosis (23). Mitosis is an important process for removing damaged mitochondria and is essential for maintaining the number and function of mitochondria in the cell. Fragmentation caused by imbalanced mitochondrial dynamics leads to further abnormalities associated with the process of cellular phagocytosis, the inability of harmful substances to be expelled from the cell, and massive neuronal death (24, 25). These changes in mitochondrial dynamics have been extensively studied in a variety of AD mouse models, the most important of which are increased expression of proteins such as DRP1 and FIS1, and a significant decrease in the expression of proteins such as Mfn1, Mfn2, Opa1, and the mitochondrial transporter protein TOM40 (26, 27). Dynamic changes in these proteins are observed in the early, middle and late stages of AD, highlighting the continuing role of mitochondrial dynamics imbalance throughout the development of AD.

**Figure 1:**
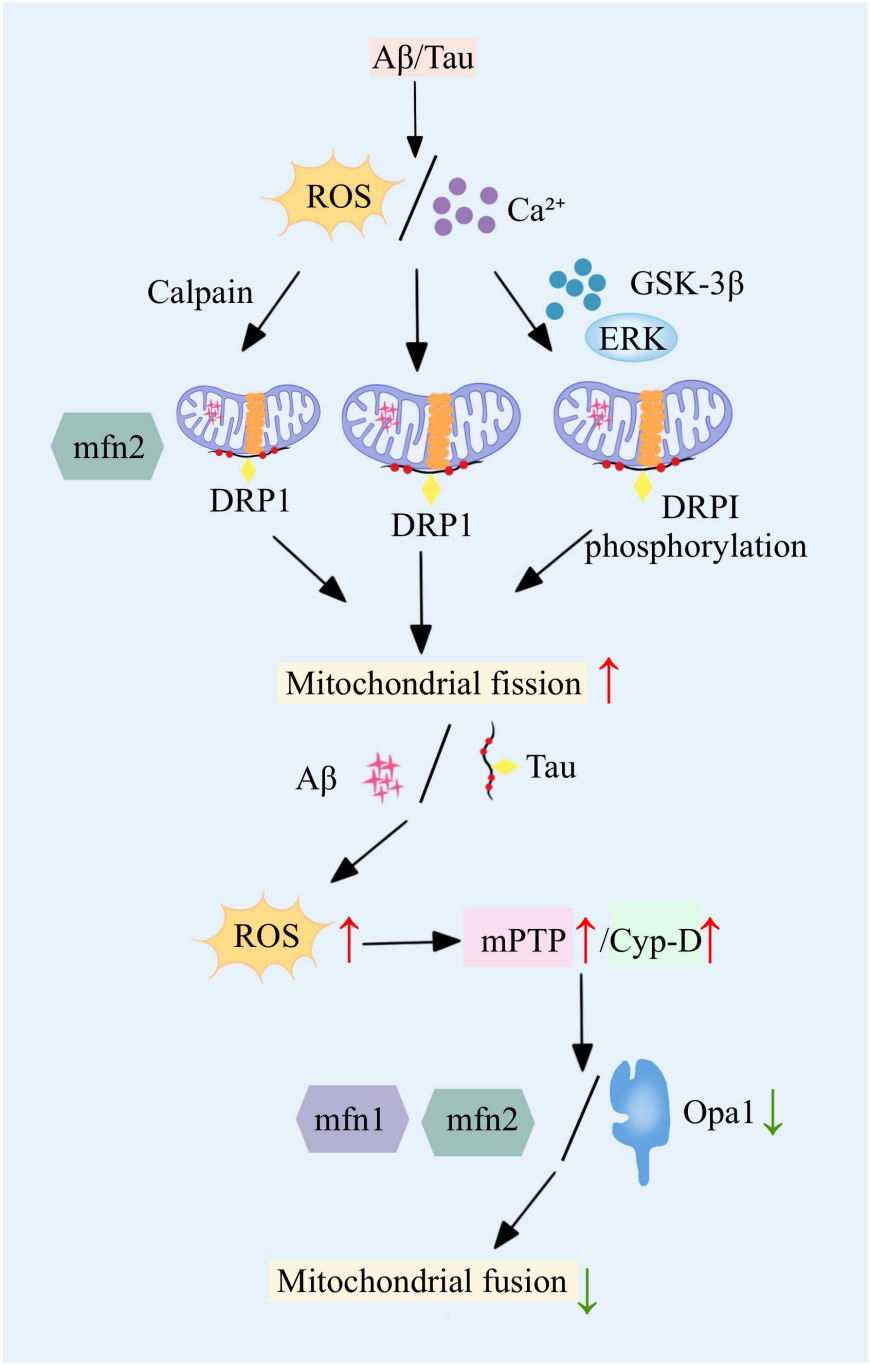
An imbalance in **Figure 1** mitochondrial dynamics drives AD onset, in which key proteins are aberrant, leading to increased fission and decreased fusion.

Imbalance in mitochondrial dynamics not only affects mitochondrial number, structure and function (**Figure 1**), but is also closely associated with disturbed neuronal energy metabolism and cell death. Therefore, the development of therapeutic strategies targeting mitochondrial dynamics may provide new ideas for the treatment of AD.

### 2.2 Imbalance of mitophagy in AD

Mitophagy mammals remove abnormal mitochondria a highly conserved cellular process. It functions through two main pathways: the ubiquitin-dependent pathway and the non-ubiquitin-dependent pathway. In the ubiquitin-dependent pathway: PTEN induced putative kinase 1(PTEN induced putative kinase 1, PINK1) and Parkin proteins have very important biological functions. When mitochondria are abnormal, PINK1 will be stalled on the outer mitochondrial membrane. At this time, PINK1 activates Parkin by phosphorylation, which in turn catalyzes the ubiquitination of mitochondrial outer membrane proteins, and the ubiquitin chain is in turn phosphorylated by PINK1. Extensively phosphorylated proteins continuously emit “eat me”, promoting mitochondrial autophagy. The non-ubiquitin-dependent pathway is dominated by specific mitophagyreceptors, such as NIX and BNIP3, which directly bind to autophagy-associated proteins through their LC3 interacting regions to initiate autophagy. Together, these two pathways ensure efficient clearance of damaged mitochondria. In AD, dysregulation of mitophagy involves a variety of specific signaling pathways and affects the expression of a wide range of genes and proteins. Accumulation of Aβ proteins and phosphorylated tau (phosphorylated tau, p-tau) in AD leads to severe abnormalities in mitochondrial autophagy, which, in turn, leads to neuronal damage. Mitophagy imbalance is a very complex process that involves not only an imbalance in mitochondrial dynamics, but also leads to calcium disruption and disruption of normal metabolic pathways (28). The high expression of Aβ and tau inhibited the targeting of PINK1 and PARK2 to the mitochondria, and reduced the aggregation of PINK1 and PARK2 to the mitochondria, which led to the inhibition of mitophagy and severe neuronal damage. inhibition and severe neuronal damage (29, 30). The accumulation of Aβ interferes with this process and hinders the clearance of damaged mitochondria. *In vitro* application of Aβ1-42 triggers mitophagy damage, as evidenced by a decrease in the ratio of PINK1, Parkin, Bcl-1, and LC3-II/I, as well as the accumulation of p62 (31, 32). In contrast, prolonged application of Aβ1-42 induces an increase in these markers, suggesting that the later stages of the mitochondrial autophagic process are blocked. In addition, lysosome-autophagosome fusion is also blocked, suggesting that Aβ1-42 leads to a normal early initiation of mitochondrial autophagy, but the later steps are hindered (33, 34). Second, p-tau affects mitochondrial transport and distribution, reducing the normal distribution of mitochondria in neurons and deteriorating energy metabolism and cellular function. Studies have shown that the 20-22 kDa Tau fragment (located between amino acids 26 and 230 of the longest human Tau isoform) can stimulate mitochondrial degradation through autophagy (35). When cellular stress causes damage to mitochondria and their inner membrane potential is depolarized, the mitochondrial potential (ΔΨm) is lost, which leads to stabilization of the PINK1 protein at the outer mitochondrial membrane. At this site, PINK1 will phosphorylate the Mfn2 protein and activate the ubiquitin-proteasome system (ubiquitin-proteasome system, UPS). Initiation of this system leads to the recruitment of Parkin protein to the outer mitochondrial membrane. With the arrival of Parkin, the damaged mitochondria are further propelled to be surrounded by an encapsulated or segregated membrane, resulting in the formation of mitochondrial autophagosomes, which are ultimately sent to the lysosome for degradation in APOE4 is associated with an early stage of mitochondrial autophagy, characterized by reduced cleavage of PINK1 and increased levels of Parkin, but appears to be blocked at a later stage, as indicated by increased levels of p62 and mitochondrial markers as indicated by an increase in p62 and mitochondrial markers (36). In addition, an imbalance in mitochondrial dynamics, particularly increased mitochondrial fission and decreased fusion, leads to mitochondrial fragmentation and impaired function. This imbalance further affects the mitophagy process, including the breakdown and recirculation of mitochondrial components in the autophagosome (37). In addition, effective removal of damaged mitochondria by autophagosomes requires precise recognition by autophagosomes and fusion with lysosomes for degradation and recycling (38). However, in AD, the accumulation of Aβ and p-tau interferes with this process by altering mitochondrial dynamics, hindering the clearance of damaged mitochondria.

**Figure 2:**
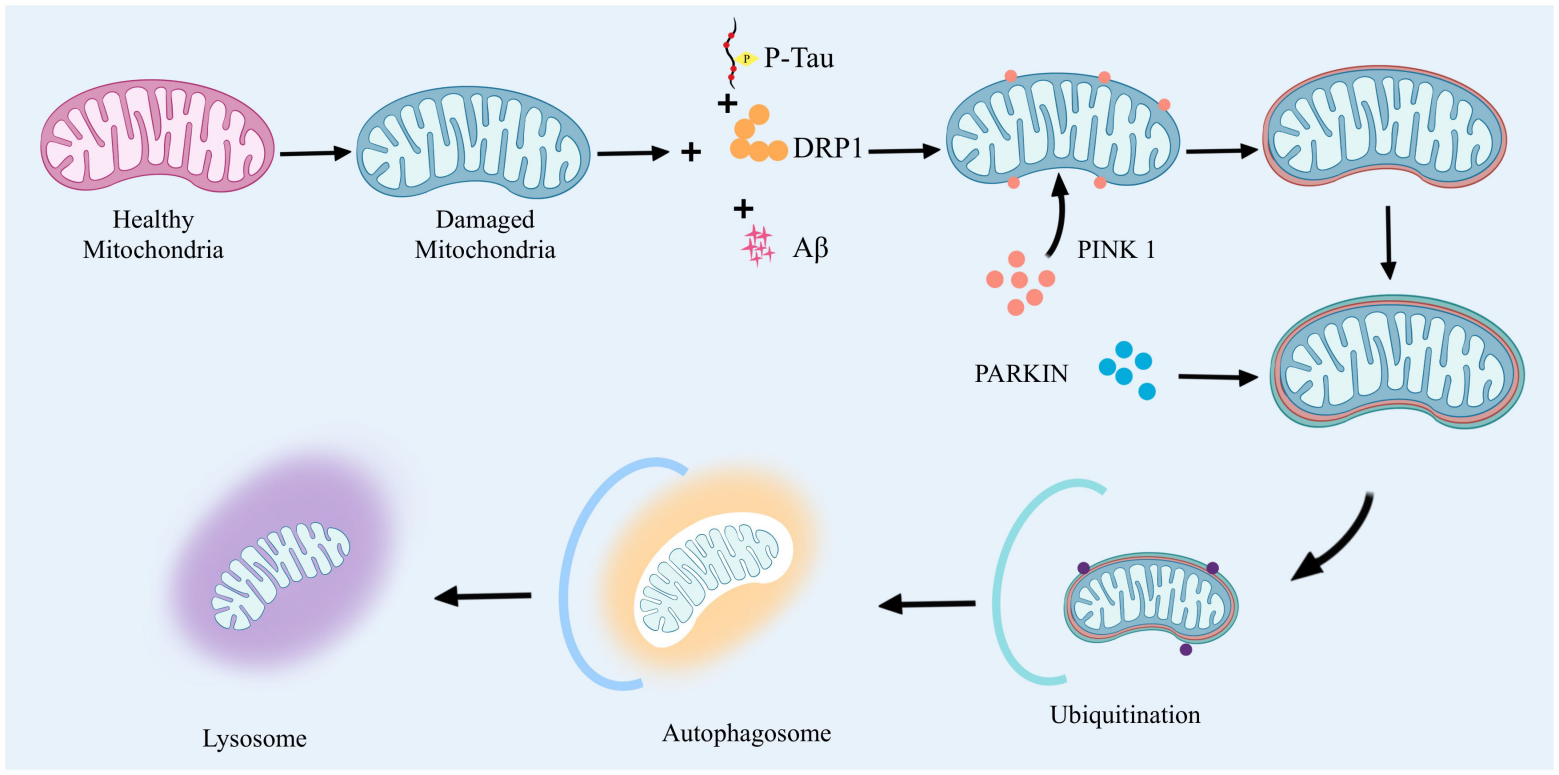
Abnormal mitophagy drives AD onset.

The imbalance of mitophagy in AD is mainly associated with the accumulation of Aβ and p-tau and affects the mitochondrial autophagic process by altering mitochondrial kinetics (**Figure 2**), which leads to mitochondrial dysfunction and neuronal damage, inducing the onset and progression of AD.

### 2.3 Dysregulation of mitochondrial calcium homeostasis in AD

In recent years, in studies of AD, it has been found that dysregulation of calcium ion homeostasis in mitochondria is closely related to cellular energy metabolism and has a huge impact on the normal function of neuronal cells. This mechanism is not only inextricably linked to cell survival and death, but also involves a series of complex biological processes (39). The level of calcium ions in mitochondria plays a key role in maintaining the homeostasis of cellular energy metabolism (40). In microglia, calcium ions help to increase the production of NADH and ATP in mitochondria by activating dehydrogenase enzymes in the tricarboxylic acid cycle, thus emphasizing the importance of controlling calcium ion levels for energy output in neuronal cells. In addition, inorganic polyphosphates (polyphosphates, polyP) have been shown to be key regulators of calcium ion homeostasis in mitochondria by controlling ca ion levels inside and outside mitochondria through control of the mPTP (41, 42). During the progression of AD, the dysregulation of mitochondrial calcium homeostasis is characterized by the mitochondria’s reduced capacity to buffer increased cytosolic calcium concentrations (43). This dysregulation leads to mitochondrial dysfunction, increased production of ROS, mtDNA mutations, and ultimately cell apoptosis, accelerating the pathological process of AD (44, 45). Notably, the neurotoxicity of Aβ in AD is closely associated with the dysregulation of mitochondrial calcium. Mitochondria become direct targets of Aβ aggregation, with its toxicity potentially mediated by disrupting calcium transfer from the endoplasmic reticulum (endoplasmic reticulum, ER) to mitochondria (46). The increased content of the mitochondrial calcium uniporter (mitochondrial calcium uniporter, MCU) elevates calcium levels within the mitochondria, revealing Aβ’s adverse effects on mitochondrial function and suggesting that blocking MCU could emerge as a potential therapeutic strategy for AD (47). The interaction between mitochondria and ER is modulated through the mitochondria-associated ER membranes (mitochondria-associated ER membranes, MAMs), which are crucial for calcium homeostasis, lipid synthesis, and mitochondrial dynamics, among other biological processes (48). In AD, enhanced MAM activity intensifies the interaction between mitochondria and ER, adversely affecting mitochondrial calcium homeostasis and further elucidating MAM’s role in the pathology of AD (49). Furthermore, the accumulation of calcium in mitochondria triggers the opening of the mPTP, a process closely linked to cell apoptosis. In AD, the abnormal opening of mPTP further impairs mitochondrial function, promoting cell death and exacerbating the pathological changes of AD (50).

The dysregulation of mitochondrial calcium homeostasis in AD reveals a complex mechanism network involving energy metabolism, Aβ toxicity, and the interaction between mitochondria and ER. These findings not only deepen our understanding of AD pathology but also provide a theoretical basis for developing new therapeutic strategies targeting mitochondrial calcium homeostasis dysregulation (**Table 1**).

**Table 1:** The role of mitochondria in AD.

Section	Key Points	Mechanisms/Effects	References
Mitochondrial Dysfunction Drives the Onset of AD	Imbalance between mitochondrial fission and fusion -Leads to mitochondrial fragmentation and dysfunction	Fission regulated by Drp1, Fis1, Mff Aβ and pTau increase fission by interacting with Drp1 and Fis1	(10–14)
Mitochondrial Dysfunction Drives the Onset of AD	Mitochondrial fragmentation and dysfunction due to increased fission and decreased fusion	Aβ induces S-nitrosylation of Drp1, promoting mitochondrial fission -Elevated calcium ions and ROS induce kinases like GSK-3 and ERK to phosphorylate Drp1	(15–18)
Mitochondrial Dysfunction Drives the Onset of AD	Impaired mitochondrial fusion due to dysfunction of fusion proteins	Fusion regulated by Mfn1, Mfn2, Opa1 In AD, fusion protein function impaired, reducing fusion	(19–22)
Mitochondrial Dysfunction Drives the Onset of AD	Disruption of ATP production and calcium regulation	Affects mitochondrial DNA integrity, ATP production, calcium regulation	(23–25)
Imbalance of Mitophagy in AD	Dysregulation of mitophagy pathways	Mitophagy: removal of abnormal mitochondria via ubiquitin-dependent and non-ubiquitin-dependent pathways	(28–30)
Imbalance of Mitophagy in AD	Impaired mitophagy due to Aβ and pTau accumulation	Ubiquitin-dependent: PINK1 and Parkin roles Non-ubiquitin-dependent: NIX, BNIP3 initiate autophagy	(31–34)
Imbalance of Mitophagy in AD	Hindered clearance of damaged mitochondria	Effective removal requires autophagosome recognition and lysosome fusion Aβ, pTau hinder clearance process	(35, 51, 52)
Imbalance of Mitophagy in AD	Further impairment of mitochondrial function and neuronal damage	Mitochondrial dynamics imbalance affects mitophagy Increased fission, decreased fusion impair mitophagy	(36–38)
Dysregulation of mitochondrial calcium homeostasis in AD	Impact on cellular energy metabolism and neuronal function	Dysregulation of mitochondrial calcium homeostasis impacts cellular energy metabolism and neuronal function	(39–42)
Dysregulation of mitochondrial calcium homeostasis in AD	Calcium ions regulate energy metabolism in mitochondria	Calcium ions in mitochondria maintain cellular energy metabolism homeostasis Inorganic polyphosphates regulate calcium ion levels via mPTP	(43–45)
Dysregulation of mitochondrial calcium homeostasis in AD	Dysregulation leads to mitochondrial dysfunction, ROS production, mtDNA mutations, apoptosis	In AD, reduced mitochondrial capacity to buffer cytosolic calcium leads to dysfunction, ROS production, mtDNA mutations, apoptosis	(46, 47)
Dysregulation of mitochondrial calcium homeostasis in AD	Aβ disrupts calcium transfer from ER to mitochondria Enhanced MAM activity and mPTP opening further impair mitochondrial function	Aβ disrupts calcium transfer from ER to mitochondria, increases MCU content, and affects mitochondrial function Enhanced MAM activity affects calcium homeostasis and mitochondrial dynamics Accumulated calcium triggers mPTP opening, linked to apoptosis	(48–50)

## 3 Mitochondria-related omics reveal the application of mitochondrial dysfunction in AD

### 3.1 Bulk-RNA-seq

Bulk-RNA-seq studies have revealed the crucial role of mitochondria and their associated pathways in the progression of Alzheimer’s Disease (AD). Transcriptomic analyses of brain cells from AD patients and age-matched control groups have shown a close association between AD pathology and mitochondrial function (53). Notably, a meta-analysis has identified seven genes that are consistently differentially expressed across all regions of the AD brain, including the early response gene ZFP36L1, RERE, PURA, OGT, SPCS1, SOD1, and NDUFS5 (54). The expression patterns of these genes in AD suggest the importance of mitochondrial function in the disease, with three of these genes (NDUFS5, SOD1, and OGT) being directly involved in mitochondrial functions (55). The NDUFS5 gene, as part of mitochondrial complex I, might impact ATP production due to its decreased expression in AD, affecting cellular energy supply. The SOD1 gene, involved in the antioxidative mechanism, helps detoxify ROS, crucial in the ETC (electron transport chain) process. In AD, downregulation of SOD1 leads to the inability of neuronal cells to effectively inhibit lipid peroxidation caused by excessive mitochondrial ROS levels, which further exacerbates neuronal cell injury and ultimately promotes the onset and progression of AD (55). OGT, a key regulatory gene encoding glycosyltransferase, plays a critical role in the mechanism of AD genesis by participating in the post-translational modification of neuronal tau and amyloid precursor protein. AD genesis mechanism. In AD, OGT is responsible for the addition of O-GlcNAc, and O-GlcNAc glycosylation promotes the expression of phosphorylated tau proteins, which in turn leads to the loss of function of microtubule proteins in neuronal cells. Interestingly, increased levels of OGT expression inhibit tau phosphorylation, while genetic downregulation of OGT in turn increases tau phosphorylation (56). Another study using RNA-seq found that gender may affect the expression of AD-related genes. Mitochondrial metabolism-related pathways are enriched in both males and females, but specific pathways such as synaptic transmission and neuronal projection are more enriched in females (57). Therefore, in the treatment of AD, great differences in personalization need to be taken into account if by targeting mitochondria-related genes and pathways. In addition, RNA-seq data based on large samples have revealed that the expression of genes such as BIN1, MAP3K3, VASP and TBC1D1 are closely associated with specific pathological features of AD. BIN1 is known to be important in maintaining normal mitochondrial function and its potential to be an effective target for AD therapy by regulating tau protein clearance and modulating neuronal activity (58, 59). The above RNA-seq studies revealed that mitochondria have multiple roles in AD, including energy metabolism, anti-oxidative stress, and regulation of the dynamic balance of Ca^2+^ (60).

Bulk-RNA-seq technology has revealed the key role of mitochondria and their related genes in AD, providing important ideas for further research on the specific mechanisms of AD and the development of targeted therapeutic strategies. By integrating data from large-scale studies and cohort studies of different ADs, we were able to re-conceptualize the mechanisms behind mitochondria-driven AD, pointing the way to the development of new therapeutic approaches.

### 3.2 scRNA-seq

Single-cell RNA sequencing (Single-cell RNA sequencing, scRNA-seq) technology has greatly contributed to our comprehensive understanding of neuronal cell heterogeneity and mitochondrial function in AD. Meanwhile, scRNA-seq technology reveals different subtypes of cell type-specific transcriptional features and changes in mitochondria-related pathways during AD progression, from which we are able to identify many key signaling molecules (61). By analyzing various types of cells in the brains of AD patients, researchers have identified significant transcriptome-specific expression differences as well as different mitochondria-associated genes and signaling pathways at different stages of AD pathogenesis (62). Mathys and colleagues et al. analyzed 48 AD patients by integrating them using single-nucleus RNA sequencing (snRNA-seq). Mathys and coworkers obtained 80,660 nuclei from the brain samples of 48 AD patients by using single-nucleus RNA sequencing (single-nucleus RNA sequencing, snRNA-seq). Colleagues, various cell types (e.g., excitatory neurons, inhibitory neurons, astrocytes, and oligodendrocytes) and their specific transcriptome expression profiles in AD were meticulously distinguished (63). This study reveals the critical role of mitochondria in different stages of AD, including differences in mitochondrial gene expression, abnormal expression of oxidative phosphorylation pathways, and dysregulation of pathways such as mitochondrial transport. First, differences in mitochondrial-related genes: the expression of the NDUFA1 and NDUFA5 genes, which are subunits of NADH dehydrogenase (part of mitochondrial complex I), as well as the subunit COX6C, a subunit of cytochrome c oxidase complex IV, were grossly aberrant, highlighting potential evidence of impaired function of the electron transport chain (electron transport chain, ETC) (64). In addition, the differential expression of SOD1 suggests that there is a significant imbalance in the antioxidant defense mechanism in AD. oXPHOS, as a core part of the ETC, any alteration in its function will directly affect the energy production of neuronal cells. scRNA-seq data directly suggest that the oXPHOS process may be impaired in AD. Further analyses showed that excitatory and inhibitory neurons are enriched for different mitochondrial pathways in early and late pathological states of AD, and that these pathways correspond to different pathological features in disease progression (65). The scRNA-seq analysis of astrocytes and oligodendrocytes showed that they play a role in AD by relying on mitochondria-related pathways (e.g., ATP metabolic pathway, detoxification response pathway, and mitophagy pathway) in the late stages of AD. And the analysis of microglia further revealed the role of mitochondrial dysfunction in AD (66). In early AD, microglia showed high expression of interferon regulatory genes, whereas in late AD, microglia showed high expression of MHC and S100 family genes. The identification of microglia subpopulations associated with amyloid-β and tau pathology suggests that these cells exhibit unique phenotypic and metabolic characteristics, highlighting the complexity of microglia and their impact on AD disease progression.

scRNA-seq technology has been able to greatly elucidate the mechanism of interaction between mitochondrial dysfunction and AD occurrence and progression. These findings not only deepen our understanding of the complex pathology of AD, but also provide potential new targets for the development of therapeutic strategies for specific cell types and disease stages.

### 3.3 Epigenomics and multi-omics

Epigenetics plays a crucial role in unraveling the mechanisms behind mitochondrial dysfunction and disease progression in AD. Recent multi-omics studies integrating epigenomics, transcriptomics and proteomics have provided new insights into the molecular pathways of AD (67). These studies found that the expression of genes associated with mitochondrial respiration and oxidative phosphorylation was highly down-regulated, whereas genes associated with AD transcription and chromatin accessibility were significantly up-regulated, suggesting that mitochondrial function in AD may be subject to complex epigenetic regulation. Epigenetic Markers and AD: Proteomic analysis has particularly highlighted an overall increase in the levels of histone acetylation (such as H3K27ac and H3K9ac) in AD, directly related to active transcription (68). Chromatin immunoprecipitation sequencing (Chromatin immunoprecipitation sequencing, ChIP-seq) analysis confirmed that in the genomes of AD patients, there are more peaks associated with H3K27ac and H3K9ac, with a significant increase in acetylation, suggesting significant changes in chromatin state in AD, which could lead to alterations in gene expression patterns (69). Functional Pathways and AD: Functional pathways associated with disease-specific gains of H3K27ac and H3K9ac include Gene Ontology (GO) terms related to transcription and nucleic acid metabolism, implying that these epigenetic modifications play crucial roles in regulating the expression of AD-related genes. DNA motif enrichment analysis further shows that transcription factors NRF1 and CTCF are enriched at sites with disease-specific gains of H3K27ac or H3K9ac, highlighting their potential role in the regulation of mitochondrial gene expression in AD (70). The Connection between GWAS and the Epigenome: Variants associated with AD identified by Genome-Wide Association Studies (GWAS) are predominantly located in non-coding regions, expected to influence disease progression through changes in transcription factor binding and regulatory element function. These changes have a high degree of cell-type specificity, especially with AD SNP enrichment significantly increased in microglia but not in neuronal subtypes, further confirming the unique role of microglia in AD pathology (71). Single-Cell Chromatin Accessibility Analysis: The research by Corces et al. using single-cell chromatin accessibility assays revealed brain regional and cell-type-specific epigenomic heterogeneity in AD. This detailed analysis provided a comprehensive map of epigenetic changes in AD, pointing to disease-related alterations in specific cell types and brain regions (72).

Multi-omics research has revealed the complex relationship between mitochondrial dysfunction and disease progression in AD, emphasizing the key role of epigenetics in regulating the expression of mitochondrial-related genes (**Table 2**). By meticulously analyzing epigenetic information, functional signaling pathways, GWAS-identified genetic variants, and chromatin accessibility data in AD, we were able to probe the mystery behind the pathological features of AD in terms of epigenetic regulation, providing new targets and ideas for future targeted therapeutic strategies. These groundbreaking findings not only deepen our understanding of the specific mechanisms behind mitochondrial dysfunction in AD, but also emphasize the importance of cell-specific epigenetic changes in disease onset and progression, and provide an important theoretical basis for further exploration of the complex pathology of AD.

**Table 2:** Application of multi-omics techniques in AD.

Section	Key Findings	Implications	Challenges	Ref
Bulk-RNA-seq	Revealed crucial role of mitochondria in AD progression.	Points to mitochondrial dysfunction as a key factor in AD.	Validation of identified genes in larger cohorts needed.	(42)
Bulk-RNA-seq	Meta-analysis identified genes with differential expression in AD.	Suggests potential therapeutic targets within mitochondrial pathways.	Translating gene expression changes to functional outcomes.	(43)
Bulk-RNA-seq	Highlighted the association between AD pathology and mitochondrial function.	Underlines the importance of mitochondrial health in AD prevention.	Dissecting cell type-specific contributions to AD pathology.	(44)
scRNA-seq	Revealed transcriptional characteristics of cell type-specificity in AD.	Suggests mitochondrial dysfunction as a target for cell-specific therapy.	Understanding stage-specific mitochondrial dysfunction.	(50)
scRNA-seq	Identified changes in mitochondrial-related pathways during disease progression.	Indicates potential for stage-specific therapeutic strategies.	Integrating epigenomic data with functional outcomes.	(53)
scRNA-seq	Differentiated cell types and expression traits emphasizing mitochondrial function’s role.	Highlights the need for detailed understanding of cell type contributions.	Identifying direct causal relationships between epigenetic changes and AD.	(54)
Epigenomics and multi-omics	Highlighted complex epigenetic regulation of mitochondrial function.	Suggests epigenetic regulation as a potential therapeutic target.	Epigenetic marker identification and validation.	(58)
Epigenomics and multi-omics	Showed significant changes in chromatin state potentially leading to gene expression alterations.	Implies targeting epigenetic markers could modulate disease progression.	Integrating multi-omics data for a holistic disease understanding.	(60)
Epigenomics and multi-omics	Linked disease-specific gains of H3K27ac and H3K9ac to functional pathways in AD.	Points to the importance of cell-specific epigenetic changes in AD.	Developing targeted therapies based on epigenetic regulation.	(61)

## 4 From a pharmacological perspective: targeting mitochondria for AD treatment

Taking mitophagy are the key target, pharmacological methods are used to improve or restore mitochondrial function, thus delaying or reversing the pathological process of AD. Here are several main pharmacological directions that act on mitophagy through distinct mechanisms, showing potential in the treatment of AD: Increasing Intracellular NAD+ Levels: Elevating intracellular NAD+ can enhance the activity of SIRT1 and SIRT3, two Sirtuin proteins, thus improving the bioenergetics of neuronal mitochondria (73). For instance, supplementation with nicotinamide, a precursor of NAD, has been shown in the 3xTgAD mouse model to enhance SIRT3 activity, ameliorate Aβ and Tau pathology, and improve learning and memory deficits. This mechanism involves enhancing mitochondrial resistance to oxidative stress, upregulating autophagy, and activating the PI3K-Akt, MAPK/ERK1/2 signaling pathways (74).Enhancing Mitochondrial Autophagy: Mitochondrial autophagy, or mitophagy, is crucial for maintaining mitochondrial health by removing damaged mitochondria to prevent AD-related mitochondrial function decline (75). Drugs that induce mild bioenergetic stress or inhibit the mTOR pathway, such as the mitochondrial uncoupler DNP and the mTOR inhibitor rapamycin, have been shown to activate autophagy and preserve neuronal function in AD-related animal models (76). Pharmacological Interventions: Specific compounds like nicotinamide riboside, Urolithin A (Urolithin A, UA), Actinonin (Actinonin, AC), and NAD+ enhancers (nicotinamide riboside, nicotinamide mononucleotide) promote mitophagy and improve AD pathology through different mechanisms. These compounds activate the PINK1/Parkin-dependent mitophagy pathway, reducing Aβ burden, improving memory deficits, restoring mitochondrial morphology and function, and increasing synapse numbers (77). Nicotinamide and Nicotinamide Riboside: These compounds, by boosting intracellular NAD+ levels, directly influence the activity of Sirtuin proteins, particularly SIRT1 and SIRT3 (78). SIRT1 and SIRT3, key regulators of mitochondrial health, improve mitochondrial bioenergetics and enhance resistance to oxidative stress through deacetylation of critical mitochondrial proteins. In AD models, these compounds activate SIRT1 and SIRT3, reducing the aggregation of Aβ and Tau proteins, improving neuron survival and function (79). They also promote the autophagy process, helping to remove damaged mitochondria and protein aggregates, thereby mitigating AD neuropathology. UA: UA, a natural compound produced by gut microbial metabolism of ellagic acids, promotes mitophagy by activating the PINK1/Parkin pathway. UA can delay the progression of AD by improving mitochondrial quality and facilitating the clearance of damaged mitochondria (77). In a mouse model of AD, treatment with UA Similar results were also observed, with reduced Aβ deposition and decreased Tau phosphorylation in mouse neuronal cells, and significant improvement in memory deficits in mice. Additionally, UA improves mitochondrial morphology, increases synaptic density, reduces inflammation, and promotes neuroprotection (80). AC: Similar to UA, AC restores mitochondrial morphology and function by activating the mitophagy pathway. AC can prevent memory deficits in AD models and reduce Aβ burden (81). AC treatment not only improves mitochondrial morphology and function but also increases the number of synapses, stimulates the clearance of Aβ plaques, and reduces neuroinflammation. It has been shown that AC exerts neuroprotective effects by attenuating pathological changes in AD while promoting mitophagy in microglia (82). Natural compounds (e.g., melatonin, trehalose, resveratrol, bexarotene, tetrahydroxy stilbene, β-asarone, etc.) are able to greatly alleviate the progression of AD by stimulating mitochondrial autophagy, clearing damaged mitochondria and activating the PINK1/Parkin pathway, reducing oxidative stress and mitigating mitochondrial damage.

The pharmacological strategies targeting mitophagy are aimed at restoring or maintaining normal mitochondrial function and reducing the occurrence of AD-related neurodegenerative diseases through various mechanisms.

## 5 Immune cell crosstalk and immunotherapy in AD

In AD, abnormalities in immune cell function also directly drive the onset and progression of AD. First, Aβ production and deposition occurs in AD. Aβ then accumulates in large numbers in the brain and begins to drive microglial cells in the brain. Microglia, as one of the most prominent immune cells in the brain, inhibit the excessive accumulation of Aβ by recognizing it and removing these abnormal proteins. However, prolonged and massive accumulation of Aβ eventually leads to microglial over-activation and the release of large amounts of inflammatory factors, which further exacerbate neurological damage (83, 84).

Immunotherapy for AD: Currently, it can be mainly categorized into two therapeutic strategies: active immunotherapy and passive immunotherapy. Among them, active immunotherapy mainly involves the study of immunization vaccines. AN1792: AN1792 is a synthetic passive immunization vaccine based on full-length Aβ protein and using QS-21 as an adjuvant. It promotes an immune response primarily by facilitating antigen presentation and activating specific T and B cells to produce antibodies that eliminate Aβ (85). These antibodies recognize and bind to aberrant Aβ proteins, labeling them so as to enhance the ability of microglial cells to remove Aβ proteins by phagocytosis.AN1792, the first clinically tested anti-Aβ vaccine, has not been particularly well-tested in clinical trials. A small number of patients develop T-cell mediated meningitis and are found to have low antibody concentrations and suboptimal therapeutic efficacy during long-term follow-up (86). CAD106: CAD106 targets the N-terminal fragment of the Aβ protein (Aβ1-6), primarily to avoid triggering a T-cell-mediated immune response against Aβ, and instead targets only B-cell-mediated antibody production.CAD106 mediates a long-term antibody response, aimed at sustained clearance of Aβ protein without activating harmful T cells (87).

Passive immunotherapy primarily utilizes monoclonal antibodies. Aducanumab: Aducanumab is a monoclonal antibody against Aβ aggregates. It has a high affinity for binding to specific epitopes of Aβ aggregates and recognizes not only formed fibers but also oligomers in the early stages of formation (88). Aducanumab promotes Aβ clearance and reduces its neurotoxicity by activating phagocytosis by immune cells through the Fc region of the antibody, particularly through interaction with the Fcγ receptor on the surface of microglial cells. Solanezumab: Solanezumab reduces aggregation and promotes clearance of circulating monomers and small oligomers by targeting the middle portion of the Aβ protein (Aβ13-28), which binds exclusively to them (89). Solanezumab reduces Aβ levels in the brain primarily by blocking the aggregation and deposition of Aβ and by transporting the antibody-Aβ complexes to the liver and clearing them. Donanemab: Donanemab is a monoclonal antibody targeting N-terminal pyroglutamate-modified Aβ. Donanemab is able to improve neurological function by binding to Aβ in plaques and subsequent antibody-mediated cellular phagocytosis, capable of decreasing perineuronal Aβ loading and ultimately directly facilitating clearance of deposited Aβ (90).

In AD immunotherapy, most of the strategies, both active and passive immunotherapy, target Aβ. from preventing Aβ formation and aggregation to directly removing formed Aβ aggregates. In the future, the development of immunotherapy will greatly facilitate the treatment of AD, improving the quality of patient survival while controlling the associated side effects as much as possible.

## 6 Conclusion

Currently, therapeutic research in AD still faces multiple formidable challenges. The first is that the understanding of AD pathogenesis is still incomplete, and there are still many unfathomable processes of occurrence and development. The second is the high failure rate of clinical trials, and the fact that some previous studies have been fundamentally misdirected. Third is the paucity of biomarkers to diagnose AD. Finally, there is a high degree of heterogeneity in AD among patients; the pathogenesis of AD is complex, involving amyloid plaques, Tau protein tangles, neuroinflammation, and metabolic disorders, and a unified pathologic model has yet to be developed. The high failure rate of clinical trials is partly attributed to the difficulty in identifying effective therapeutic targets due to these complex mechanisms, while the heterogeneity of patients also makes it difficult to standardize therapeutic regimens.

Future research directions need to be multifaceted. The first is to continuously explore the pathogenesis of AD and establish a sound mechanistic basis. The second is to develop multi-targeted drugs, which should not only target traditional amyloid plaques and Tau proteins, but also include mechanisms such as neuroinflammation, oxidative stress and mitochondrial dysfunction. Thirdly, efforts also need to be made to advance personalized therapeutic strategies, using genomics, epigenomics, and proteomics data to develop precise treatment regimens for individual patients. Finally, the development of early diagnostic biomarker tools is also critical, especially those that can detect disease before the onset of clinical symptoms, such as early detection enabled by liquid biopsies and high-resolution imaging. In addition, research into neuroprotective and neuroregenerative approaches, including stem cell therapies and gene therapies, which have the potential to make breakthroughs in preventing or reversing neuronal damage, is an important future direction. The combination of artificial intelligence and big data technologies will play an important role in the understanding of disease mechanisms, the discovery of new therapeutic targets, and the optimization of clinical trial design and patient management. Through these multifaceted efforts, the future holds the promise of overcoming current limitations and developing more effective AD treatments that will significantly improve patients’ quality of life and prognosis.

In recent years, intensive research on mitochondria has highlighted that mitochondrial dysfunction is a key centerpiece driving the pathogenesis of various neurodegenerative diseases. This review comprehensively explores the important role of mitochondrial dysfunction in the onset and progression of AD. Processes such as mitochondrial autophagy, disturbed mitochondrial dynamics, and disruption of Ca2+ homeostasis, all of which have specific mechanisms of action in AD onset and progression, are summarized in focus. Advances in mitochondrial multi-omics research have greatly improved our understanding of the complex pathomechanisms of AD, especially in identifying additional key genetic targets, signaling pathways and pharmacological interventions for treatment. By studying the molecular mechanisms of mitochondrial dysfunction and utilizing comprehensive multi-omics techniques, we have discovered an increasing number of key mechanisms that regulate the etiology and progression of AD. These findings not only reveal the critical role of mitochondria in normal neurons and disease, but also demonstrate that targeting mitochondria has significant potential in AD therapy. With further research, we have every reason to believe that by studying mitochondria and its multi-omics, we will be able to further decipher the complex molecular and cellular maps of AD in the future, which will facilitate the development of personalized and effective therapies, improve the quality of patient’s survival, and reduce the global economic and health burden.

## Author contributions

ZL: Writing – review & editing, Writing – original draft. QZ: Writing – review & editing. NR: Writing – review & editing. HZ: Writing – review & editing. XZ: Writing – review & editing, Writing – original draft.
